# Financial viability of electric vehicle lithium-ion battery recycling

**DOI:** 10.1016/j.isci.2021.102787

**Published:** 2021-06-25

**Authors:** Laura Lander, Tom Cleaver, Mohammad Ali Rajaeifar, Viet Nguyen-Tien, Robert J.R. Elliott, Oliver Heidrich, Emma Kendrick, Jacqueline Sophie Edge, Gregory Offer

**Affiliations:** 1 Department of Mechanical Engineering, Imperial College London, London, UK; 2 The Faraday Institution, Quad One, Becquerel Avenue, Harwell Campus, Didcot, UK; 3 Cognition Energy Ltd, 30 Upper High Street, Thame, Oxfordshire, UK; 4 School of Engineering, Newcastle University, Newcastle upon Tyne, UK; 5 Faraday Institution, ReLiB Project, Newcastle University, Newcastle upon Tyne, UK; 6 The Department of Economics, JG Smith Building, University of Birmingham, Birmingham, UK; 7 Faraday Institution, ReLiB Project, University of Birmingham, Birmingham, UK; 8 Tyndall Centre for Climate Change Research, Newcastle University, Newcastle upon Tyne, UK; 9 Birmingham Centre for Strategic Elements and Critical Materials, University of Birmingham, Birmingham, UK; 10 School of Metallurgy and Materials, University of Birmingham, Birmingham, UK

**Keywords:** electrochemical energy storage, energy resources, energy policy, energy application, energy systems

## Abstract

Economically viable electric vehicle lithium-ion battery recycling is increasingly needed; however routes to profitability are still unclear. We present a comprehensive, holistic techno-economic model as a framework to directly compare recycling locations and processes, providing a key tool for recycling cost optimization in an international battery recycling economy. We show that recycling can be economically viable, with cost/profit ranging from (−21.43 - +21.91) $·kWh^−1^ but strongly depends on transport distances, wages, pack design and recycling method. Comparing commercial battery packs, the Tesla Model S emerges as the most profitable, having low disassembly costs and high revenues for its cobalt. In-country recycling is suggested, to lower emissions and transportation costs and secure the materials supply chain. Our model thus enables identification of strategies for recycling profitability.

## Highlights

• Comprehensive techno-economic cost model for electric vehicle battery recycling
• Net recycling profits for recycling in Asia, Europe, and the US are compared
• Reducing transportation and disassembly costs is crucial for a profitable process
• Economies of scale and battery materials are decisive for recycling profits

## Graphical abstract

**Figure:**
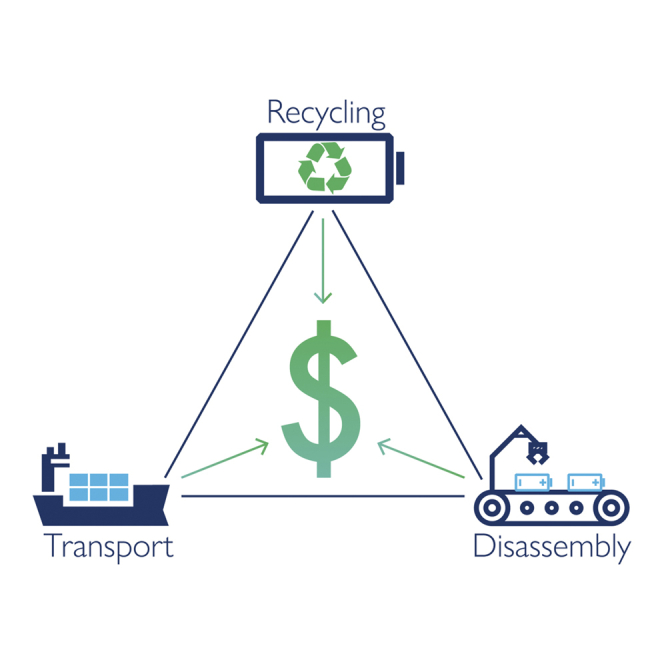

## Introduction

The decarbonization of the transport sector is a critical step in the efforts to drastically reduce global greenhouse gas (GHG) emissions (Creutzig et al., 2015; Hill et al., 2019). Electric vehicles (EVs) powered by lithium-ion batteries (LIBs) have emerged as one of the most promising options (Crabtree, 2019). In the coming decade, the LIB market is predicted to grow exponentially, due to an industry and policy push and consumer pull of EVs (International Energy Agency, 2019). As a consequence, the demand for raw materials will drastically rise (Gaines, 2018; Jones et al., 2020). This is of particular concern for the supply of critical materials such as lithium and cobalt (Critical Raw Materials Resilience: Charting a Path toward greater Security and Sustainability, 2020). Moreover, the accumulation of large amounts of waste batteries is expected (Chen et al., 2019; Harper et al., 2019). A predicted 23 million EV cars sold globally in 2030 could lead to 5,750,000 tonnes of retired batteries by 2040, assuming a battery lifetime of 10 years and 250 kg per battery pack (International Energy Agency, 2019).

**Figure 1:**
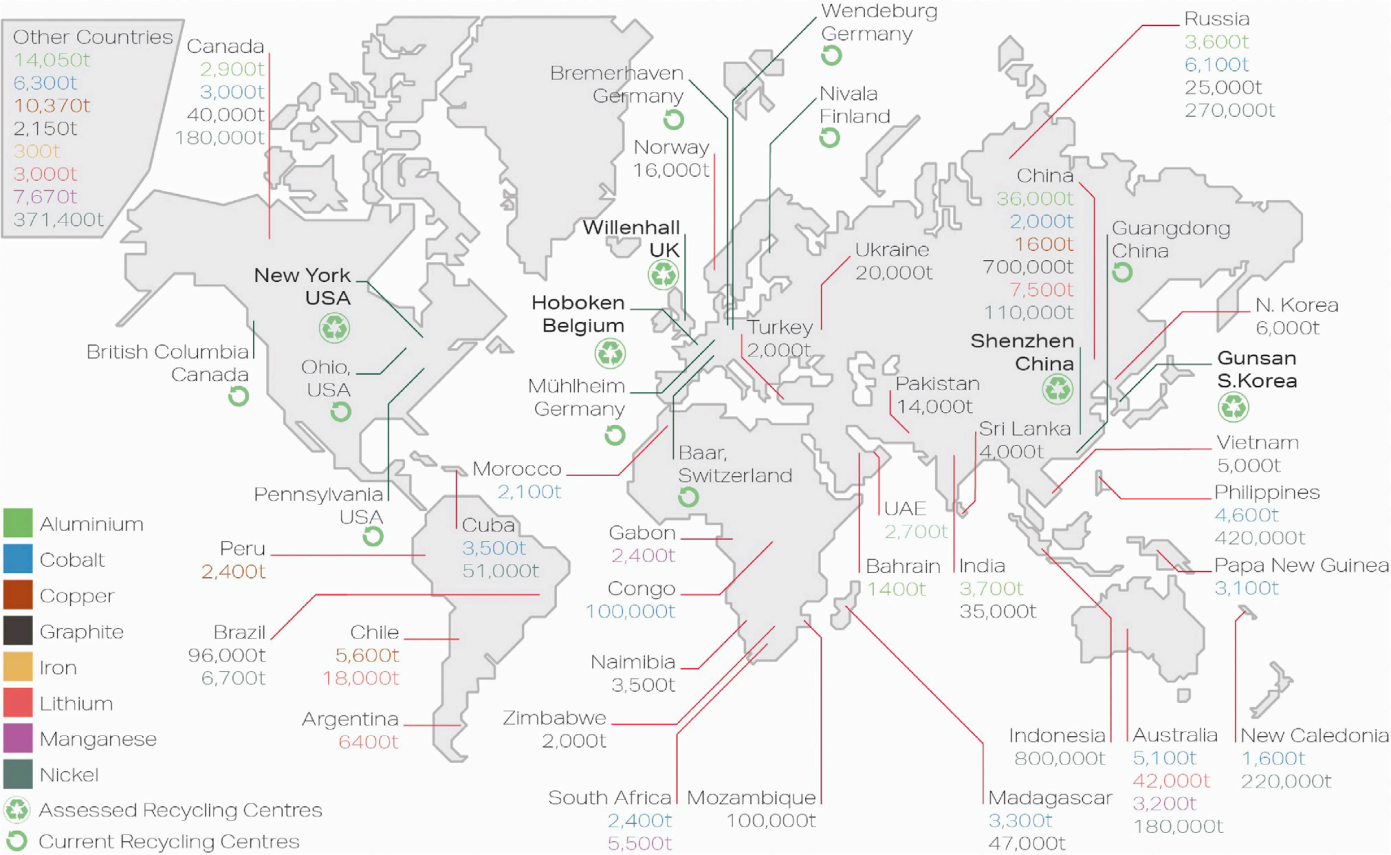
Global mining and recycling World mine production for raw materials contained in LIBs in 2019 (Mineral Commodity Summaries 2020, 2020) and locations of a selection of LIB recycling facilities. Recycling facilities shown in bold are the ones assessed in this study.

Discarding batteries into landfills might result in the leakage of toxic compounds into the environment and landfill fires caused by defective or degraded cells (Winslow et al., 2018). Instead, recycling allows for valuable metals such as cobalt and nickel to be recovered, lowering the raw material demand and securing an alternative raw materials supply chain, gaining independence from exporting economies (Figure 1) (Helbig et al., 2018; Mineral Commodity Summaries 2020, 2020; Olivetti et al., 2017; Skeete et al., 2020; Sommerville et al., 2021; Steward et al., 2019). Moreover, efficient recycling processes can help to avoid energy- and emission-intensive material processing (Ciez and Whitacre, 2019; Dunn et al., 2012; Gaines et al., 2011; Hao et al., 2017; Mohr et al., 2020; Xiong et al., 2020). Current recycling processes can be mainly classified under three types, namely pyrometallurgical, hydrometallurgical, and direct recycling (Chen et al., 2019; Dunn et al., 2012; Elwert et al., 2018; Fan et al., 2020; Gaines, 2018; Harper et al., 2019; Huang et al., 2018).

The widespread implementation of LIB recycling is hampered by insufficient recycling efficiencies, environmental impacts, safety hazards and logistical challenges, such as collection and transportation (Gaines et al., 2018; Harper et al., 2019; Yun et al., 2018). A large variety of pack designs and battery chemistries further add to the complexity of recycling (Chitre et al., 2020). Given the currently rather low number of End-of-Life (EoL) EV LIBs, recycling costs are still high and profits low, discouraging EV and battery manufacturers from pursuing the recycling of retired batteries effectively (Heelan et al., 2016; Rohr et al., 2017). It is thus crucial to identify cost-cutting/profit-increasing opportunities in the recycling process through detailed techno-economic assessments, in order to incentivize the industry to increase its recycling capacity. Indeed, to date, several techno-economic studies of LIB recycling have been published, analyzing the recycling cost of several battery chemistries and various recycling methods (Fan et al., 2020; Li et al., 2018; Samarukha, 2020; Xiong et al., 2020; Yang et al., 2018). However, most techno-economic studies are limited by either the system boundaries of their assessment (e.g. battery chemistries or excluding transportation) or geographical boundaries (e.g. transboundary movements). We provide here a global and comprehensive techno-economic framework, allowing for the attribution of a $·kWh^−1^ value for net recycling profit for different battery chemistries (i.e. LiMn_2_O_4_ (LMO), LiFePO_4_ (LFP), LiNiCoAlO_2_ (NCA) and LiNiMnCoO_2_ (NMC)), recycling processes (pyrometallurgical, hydrometallurgical, direct) and recycling locations (South Korea, China, the US, Belgium, and the UK) (Figure 1). This cost model takes into account the entire recycling chain, starting from battery collection, transportation, disassembly, recycling, and revenue generated from recovered cell and pack materials (Figure S1). Furthermore, the impact of the battery pack design on the recycling cost is illustrated using the examples of Tesla Model S, Nissan Leaf, and Porsche Taycan packs. Our study reveals current obstacles toward recycling profitability and discusses possible strategies for an economically viable recycling process, ultimately supporting the industry to apply truly circular economy principles.

## Results

The UK, as origin of the EoL LIB, with the recycling locations in Belgium, China, South Korea, and the US, were chosen to be representative of the current global battery economy, where battery use and recycling stages are often located in different parts of the world. The UK as recycling location was selected as an example for in-country recycling. The facility was placed in Willenhall, where a battery recycling plant was recently opened (Fenix to open West Midlands battery plant, n.d.).

**Figure 2:**
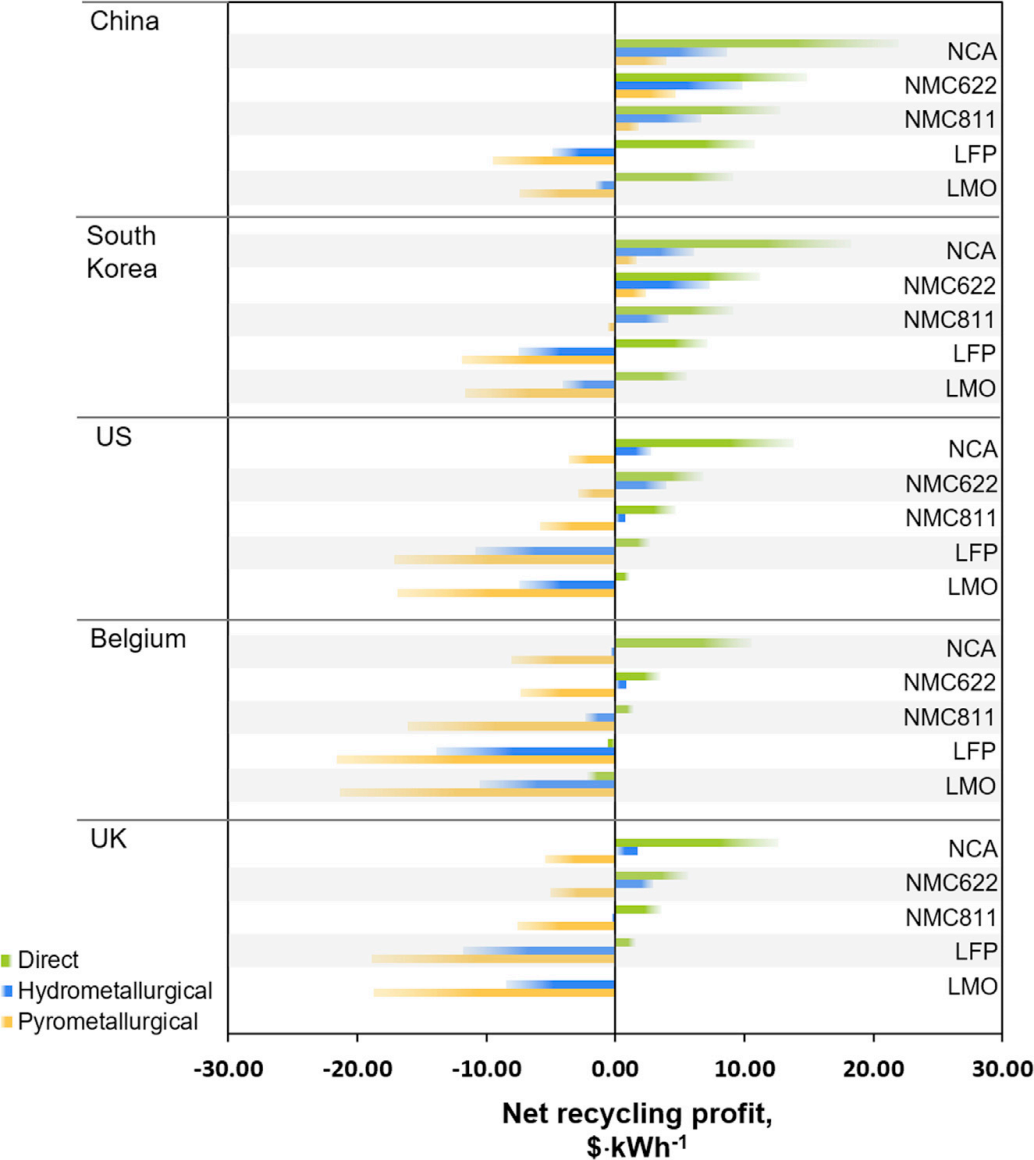
EV battery net recycling profits Net recycling profits in $·kWh^−1^, compared for five countries, using quoted transportation costs. Bars pointing to the left show an overall loss, and bars pointing to the right an overall profit.

Figure 2 and Table S1 summarize the Net Recycling Profit *NRP* (in $·kWh^−1^; Figure S2 for values given in $·kg^−1^), for a 240 Wh·kg^−1^ battery pack. Various chemistries, recycling processes and locations are compared, as calculated according to Equations (Equation 1), (Equation 2) (see supplemental information). The highest NRPs are achieved for (i) recycling in China, (ii) direct recycling, and (iii) NCA batteries. The lowest profits are obtained for: (i) recycling in Belgium, (ii) pyrometallurgy, and (iii) LMO and LFP chemistries.

To understand the origin of the cost differences, transportation, disassembly, recycling costs, and revenues will be elaborated on in the next sections, and strategies for cost reductions are discussed.

### Transportation costs

**Figure 3:**
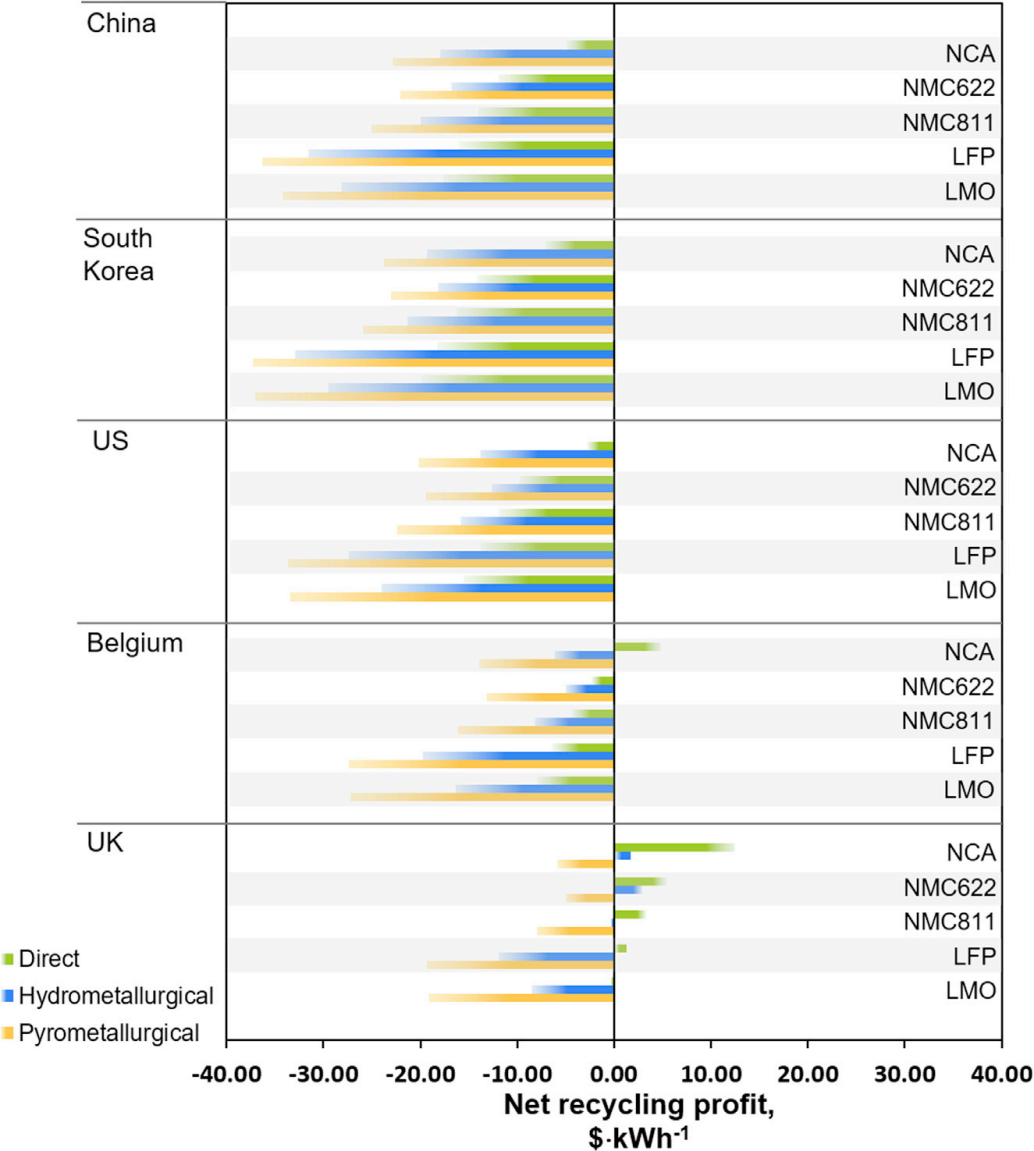
EV battery net recycling profits with increased transportation costs Net recycling profit in $·kWh^−1^ compared for five countries, assuming transportation costs as given in EverBatt (Dai et al., 2019).

Based on quoted transportation costs for 8,000 tonnes cells (still in battery packs, Figure S3A), shipping from the UK collection point to South Korea and China amounts to 1.24 $·kWh^−1^, to Belgium and the UK 0.39 $·kWh^−1^, and to the US 1.55 $·kWh^−1^. The contribution of transportation to the total recycling cost is 7–13% for China, South Korea and the US, depending on the recycling method, and ca. 2% for Belgium and the UK. When implementing the transportation fees given in EverBatt instead of the above quotes in the cost model, the overall transportation costs become significantly higher (Figure S3B). In this case, shipping to China costs 28.02 $·kWh^−1^, to South Korea 26.62 $·kWh^−1^ and to the US 18.08 $·kWh^−1^. To Belgium and the UK, transportation amounts to 6.22 $·kWh^−1^ and 0.83 $·kWh^−1^, respectively. This increases the transportation cost contribution to >70% for China, 65–70% for South Korea, 50% for the US, 20–25% for Belgium, and 5% for the UK. Here, only in-country recycling in the UK and direct recycling of NCA in Belgium become profitable (Figure 3, Table S2). Recycling abroad becomes uneconomic for all chemistries and recycling procedures. In order to make direct recycling of an NCA pack profitable in China, for instance, the transportation cost would need to drop by >60% to circa 10.50 $·kWh^−1^ (Figure S4). For other battery chemistries or for pyrometallurgical recycling, the transportation costs would need to decrease even further to achieve profitability. The difference in transportation costs between the quoted costs and EverBatt might stem from the fact that the transportation cost in EverBatt dates from 2015 and is based on the U.S. average, whereas the costs based on quotes are recent and specifically given for the transportation from collection site in the UK to the respective recycling site.

### Disassembly costs

The Tesla Model S battery pack disassembly was used as an example to calculate the disassembly cost (Equations (Equation 3), (Equation 4), (Equation 5) and Table S3). The calculated disassembly costs amount to 0.25 $·kWh^−1^ in China, 0.84 $·kWh^−1^ in South Korea, 1.68 $·kWh^−1^ in the US, 4.04 $·kWh^−1^ in Belgium, and 2.84 $·kWh^−1^ in the UK (Figure S5). The disassembly cost contribution to the total recycling cost is 2% for China, 5–7% for South Korea, 8–11% for the US, 15–21% for Belgium, and 12–17% for the UK. The cost differences between the four countries originate from the varying labor costs, Belgium having the highest (48.1 $·hr^−1^) and China the lowest (3 $·hr^−1^) costs.

### Recycling process costs

Considering the recycling process costs (i.e. labor, materials, utilities and general expenses) (Figure S6, Equations (Equation 6), (Equation 7); supplementary information), hydrometallurgy is the cheapest process; pyrometallurgy the most expensive one. The latter presents higher general expenses and utility costs and is more labor intensive leading to increased labor costs. Hydrometallurgical recycling has higher material costs, due to chemicals for leaching, for instance. The recycling costs for all processes are the lowest in China, due to lower labor costs and general expenses (Dai et al., 2019). The recycling process itself contributes 75-90% to the overall recycling costs (for the case of quoted transportation costs). Note that direct recycling is still in its early development and adoption stages for EV battery recycling. The input data in the EverBatt model might therefore be less representative of large-scale direct recycling than it is for pyro- and hydrometallurgical recycling (Dai et al., 2019), and the cost values might change in the future.

### Revenue from cell components

Overall, direct recycling leads to higher revenues compared to pyro- and hydrometallurgical processes, as more material can be recovered (Figure S7). Regarding the various chemistries, it can be concluded that the higher the cobalt content, the higher the revenue. This is due to the elevated value of cobalt (51.3 $·kg^−1^, in product form) compared to manganese (3.1 $·kg^−1^) and nickel (11.3 $·kg^−1^) (Dai et al., 2019). Besides cathode materials, copper is an important revenue source and makes up to 100% of the revenue for LFP and LMO for the pyrometallurgical process (Figure S8). Aluminum and graphite, on the other hand, contribute little to the total revenue. Revenues for pack components other than the cells have not been included in Figure S7.

## Discussion

The results presented above show that, in most cases, LIB recycling remains uneconomical and profitability is achieved only under a very narrow set of conditions of chemistry, location, and process. This bears the risk of impeding the establishment of a large enough recycling sector capable of dealing with the predicted amount of EoL batteries. Moreover, the lack of recycling profitability might lead to an increased export of EVs or batteries to countries without strong hazardous waste legislation, either legally for second-hand use or illegally (Baars et al., 2020; Green, 2017; Skeete et al., 2020; Steward et al., 2019). It has been estimated that the latter is the case for 30% of vehicles in the UK (Skeete et al., 2020). If recycling remains unprofitable, battery waste mountains could build up, which, if uncontrolled, bear a significant environmental and safety risk, as toxic chemicals could leak into the environment and landfill fires might occur (Winslow et al., 2018). Moreover, valuable materials that could be recovered and reused would simply be wasted.

In order to prevent such scenarios, two strategies can be envisaged: (i) consistent international battery legislation that requires Original Equipment Manufacturers to collect their retired batteries for free from users, encourages the extension of the battery life cycle through refurbishment or repurpose for a second life, and regulates mandatory recycling at the EoL of batteries, and (ii) making recycling an economically viable process. However, the question remains: How can LIB recycling be made profitable? In the next sections, various strategies for the increase of the profit margin are discussed.

### The impact of transportation costs

Comparing the results in Figures 2 and 3, the significant impact of transportation costs on the profitability of battery recycling becomes apparent. In this study, in-country recycling in the UK clearly outperforms overseas recycling, due to significantly reduced transportation costs, even compensating for increased labor costs in Europe compared with China, for instance. This strongly encourages the option of a vertically integrated, in-country recycling chain, analogous to vertically integrated battery manufacturing. The most pertinent advantages of in-country recycling are (i) the reduction of transportation distances and costs, (ii) the reduction of safety risks associated with the transport of degraded LIBs, (iii) a decrease of GHG emissions occurring from transportation, and (iv) securing the supply chain for critical elements. The latter is particularly significant for the growing European battery manufacturing industry and the establishment of Gigafactories, such as in the UK and Germany.

### Profitability through scale

**Figure 4:**
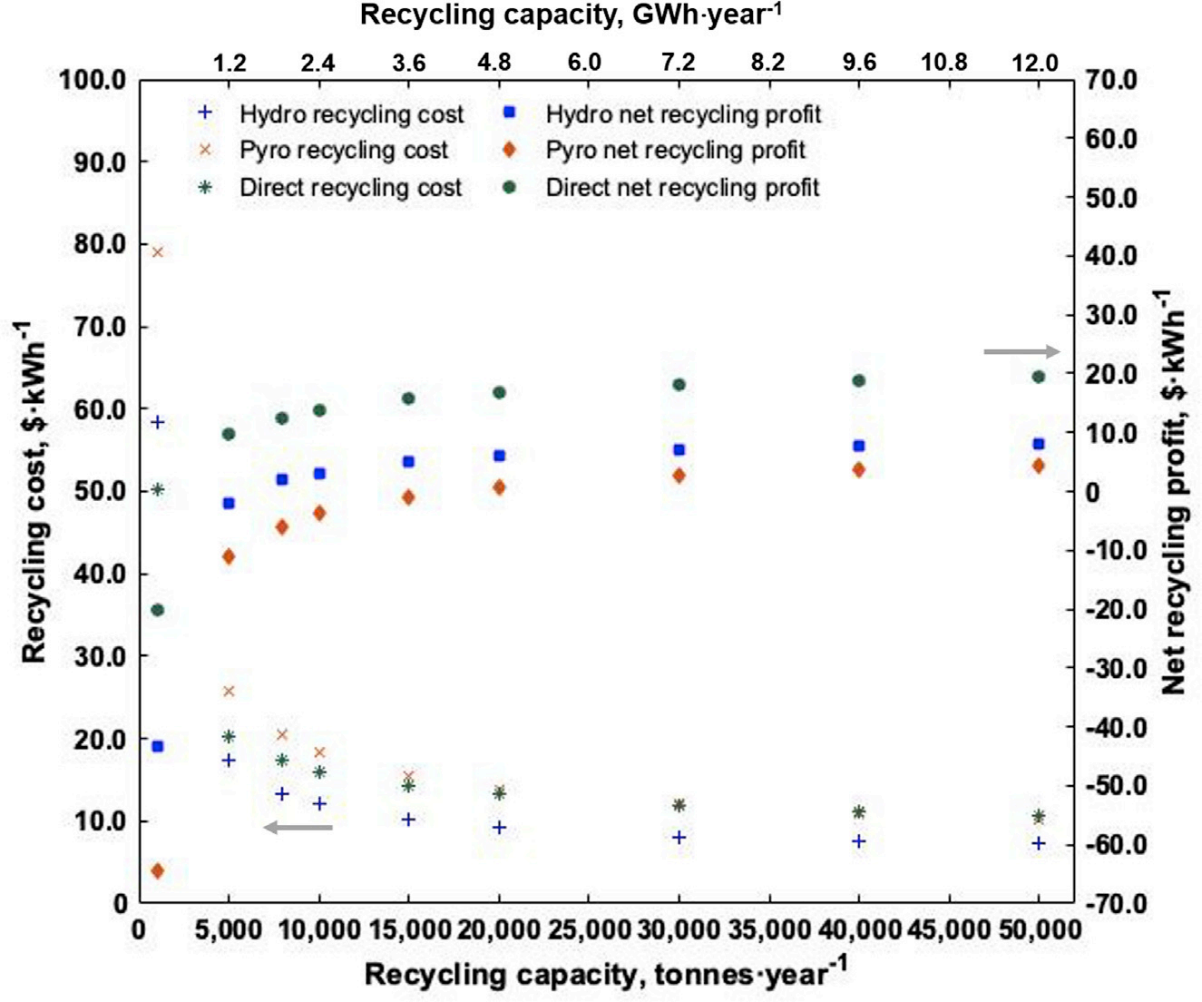
Economies of scale Recycling cost (left) and net recycling profit (right), as a function of the yearly recycling capacity in the UK for a 240 Wh·kg^−1^ NCA battery pack.

To assess the importance of economies of scale for recycling profitability, a sensitivity analysis was performed for the recycling cost and NRP, as a function of the yearly cell throughput in a UK recycling facility for an NCA battery pack (Figure 4). The estimated recycling cost decreases significantly with volumes between 1,000 and 15,000 tonnes per year. In agreement with results shown in Figure S6, the hydrometallurgical process is slightly cheaper, compared to pyrometallurgical and direct recycling. Regarding the NRP, the differences between the recycling methods are more pronounced, with direct recycling achieving the highest profit. The breakeven points for the three processes lie at 17,000 tonnes per year for pyrometallurgical, 7,000 tonnes per year for hydrometallurgical and 3,000 tonnes per year for direct recycling. However, since little industry data for direct recycling is currently available, these cost and profitability thresholds should be interpreted with care. Moreover, the here shown breakeven points for recycling profitability might vary strongly depending on the price of the recovered materials. If the price of cobalt, nickel, and lithium were to increase dramatically with the increase in demand, then a recycling plant could become profitable much sooner than we forecast. In turn, raw material prices are dependent on the investment cycle for mining companies, lags between funding and delivery of raw materials and geopolitical factors that can disrupt supply. The current trend to move away from high cobalt contents toward Ni-rich and LFP battery chemistries will have an important impact on the recycling profitability, as cobalt is the most valuable material in current battery chemistries. To illustrate this, Figure S9 compares economies of scale for an NCA battery with and without revenue generated from recovered cobalt. In this scenario, the profitability threshold increases to >50,000 tonnes per year for pyrometallurgical and circa 17,000 tonnes per year for hydrometallurgical recycling.

### Policy intervention

The difference in the breakeven points between the two scenarios (revenue and no revenue from Co) emphasizes the sensibility of recycling profitability. Although our analyses suggests that domestic recycling will become profitable in the foreseeable future, it will take time for the sector to scale up and the latecomers would be threatened by existing international players who may be able to more quickly benefit from economies of scale. The high cost barrier to establish a recycling facility, due to high recycling process costs (Figure S6), combined with the uncertainty in domestic EV adoption rates and recycling demand introduces further challenges for firms looking to invest in this sector. Also the amount and location of recycling plants in a country will influence the profitability (Nguyen-Tien et al., n.d.). It is also important to remember that many of the benefits from domestic recycling would not necessarily translate into profits. For example, security of raw material supply for domestic EV manufacture and the environmental benefits from reduced energy use and emissions. If a government is keen to secure these benefits and support the sector more broadly it is likely that some sort of intervention will be needed, at least during the early years of the industry. Policy support could include, for example, (i) a strong and credible commitment to the electrification of transportation, (ii) strict enforcement and regulation of end-of-life of EVs, and (iii) subsidies, tax relief and other financial incentives to encourage the development of a recycling sector. The latter is crucial for an early setup and operation of local recycling activities when recycling demand does not reach a viable level yet.

### Impact of pack design

Simplifying battery pack disassembly is an important step in reducing recycling costs, especially for countries where labor costs are high. Currently, EV batteries are disassembled manually, requiring a significant amount of labor time. Since disassembly cost depends on the number of steps required to dismantle a pack to its constituent cells, we were interested in the impact of the specific pack design. Based on publicly available information and battery teardowns for the Tesla Model S, Porsche Taycan, and Nissan Leaf, an approximate number of disassembly steps was derived (Tables S3–S5) and the disassembly costs (in $·kWh^−1^) calculated (Equations (Equation 3), (Equation 4), (Equation 5)). As shown in Figure S10, the Nissan Leaf pack has the highest disassembly cost, followed by the Taycan; the Tesla pack is the least cost-intensive due to its reduced amount of parts.

In order to reduce disassembly costs, packs should be “designed for disassembly” (Schwarz et al., 2018; Thompson et al., 2020). One strategy, as shown here, is the reduction of the pack components (e.g. modules) that need to be taken apart. Taking into account the requirements for streamlined EoL processes during the battery design and beginning of life manufacturing phases will allow the optimization of disassembly processes and thus reduce costs. In addition, a partially or fully automated disassembly process could decrease disassembly costs (Harper et al., 2019; Wegener et al, 2014, 2015).

### Recycling cost of commercial battery packs

To facilitate direct comparison, in the above cost calculations a uniform battery design with the same energy density (240 Wh·kg^−1^) was assumed. However, in reality, a large variety of distinct battery packs are implemented, depending on the battery and car manufacturers. This renders not only recycling more complex and difficult to standardize, but also makes general recycling cost predictions more complicated. The latter are, however, important for battery manufacturers and the recycling industry, in order to be able to estimate additional costings and predict profits. We therefore performed a techno-economic analysis of a variety of commercial battery packs, using the model described in Equations (Equation 1), (Equation 2), (Equation 3), (Equation 4), (Equation 5), (Equation 6), (Equation 7), (Equation 8), (Equation 9), (Equation 10), (Equation 11). As examples, the Tesla Model S (NCA), Porsche Taycan (NMC622) and Nissan Leaf (LMO) packs were chosen. In order to account for future battery technology developments, a theoretical Tesla Model S pack using an LFP cathode and a Porsche Taycan with an NMC811 chemistry were also modeled. The battery specifics for each pack are summarized in Table S6. Transportation costs are based on agency quotes (Figure S3).

**Figure 5:**
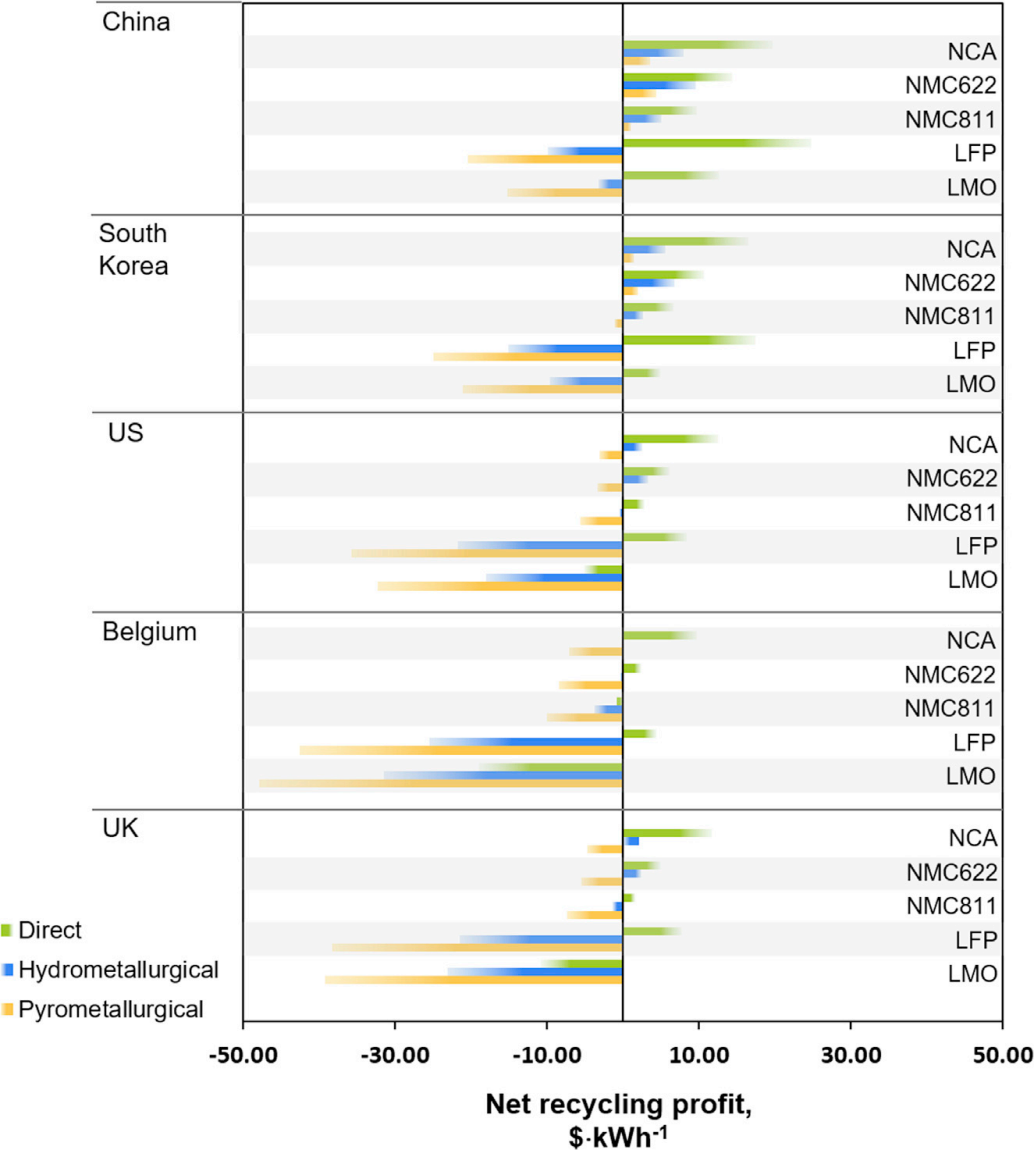
Net recycling profits for commercial EV battery packs Net recycling profit in $·kWh^−1^ for the recycling of various commercial battery packs.

Figure 5 and Table S7 summarize the NRP for the battery packs, processed via pyrometallurgical, hydrometallurgical, and direct recycling in the selected countries, with the collection point in the UK. In analogy to the 240 Wh·kg^−1^ battery pack recycling (Figure 2), direct recycling gives the highest NRP, pyrometallurgy the lowest. Recycling in China is the most profitable. The outcomes show that energy density becomes a decisive factor and can significantly influence the values on a kWh basis. Comparing a uniform battery pack, LFP and LMO chemistries achieved the lowest NRP (Figure 1) due to their lower revenues, compared to cobalt-containing materials (Figure S7). Here, however, for direct recycling, the theoretical Tesla LFP pack is comparable to the Tesla NCA one, both having the highest NRP in $·kWh^−1^. This can be explained by the lower energy density of the LFP pack (108 Wh·kg^−1^), in turn increasing the revenue per kWh. Note, however, that for pyro- and hydrometallurgical recycling, LFP remains more cost-intensive compared to the other battery chemistries, as no valuable materials besides copper are recovered.

### Conclusions

In this paper, a comprehensive techno-economic analysis of the recycling process for EV LIBs is presented. The NRP is discussed with regards to transportation, disassembly, and recycling costs, as well as the revenue generated from recovered materials. It is shown that financially viable recycling can be achieved via (i) recycling in locations with low labor and fixed costs such as in China, which reaches an NRP of up to 21.91 $·kWh^−1^, (ii) the reduction of transportation costs, where in-country recycling reduces costs by up to 70% (from 1.24 $·kWh^−1^ for UK-China to 0.39 $·kWh^−1^ for UK-UK), (iii) economies of scale, (iv) recycling of high value battery chemistries such as NCA, (v) direct instead of pyrometallurgical recycling, and (vi) the development of easy to disassemble battery packs. Moreover, policy interventions in the form of subsidies and other financial means might be necessary to ensure a profitable process. This becomes especially essential in the first operating years of a recycling plant until economies of scale are reached or when high value materials such as cobalt are not included anymore in future battery chemistries. Assessing the NRP of various commercial battery packs shows that the Tesla Model S has higher recycling profits (12–19 $·kWh^−1^ depending on the country) compared to other battery packs. This can be explained by the reduced disassembly cost, which is 80% lower than a Nissan Leaf pack, for example, and by the increased revenue for NCA. This study provides a global techno-economic assessment framework of battery recycling processes, which revealed the economic weaknesses of current recycling and presented opportunities for the development of a profitable future LIB recycling industry.

### Limitations of the study

Our work presents a comprehensive model for the calculation of the recycling cost and profit for various EV battery chemistries in different countries via different recycling processes. Transportation and disassembly costs have been determined based on specific assumptions and might vary depending on the shipping company and chosen disassembly process, respectively. Also, the materials value can fluctuate significantly over time, which has not been taken into account. Furthermore, since the direct recycling process is still relatively new, only little cost input data is available so far. With more experience in the future with this process, the cost input values can be adjusted and the final net recycling profit for direct recycling can be updated.

## STAR★Methods

### Key resources table

**Table:** 

REAGENT or RESOURCE	SOURCE	IDENTIFIER
**Bacterial and virus strains**		

Biological samples		

**Software and algorithms**		

EverBatt 2019	Dai et al. (2019)	https://www.anl.gov/egs/everbatt

**Other**		

Transportation quotes	DHL	https://app.mydhli.com/get-a-quote?utm_source=quote.mydhli.com&utm_medium=referral&utm_campaign=redirect
Labor cost Belgium and UK	Kolakovic and Lethyienne (2020)	https://ec.europa.eu/eurostat/documents/2995521/10624905/3-31032020-BP-EN.pdf/055df0e0-980d-27b9-a2a9-83b143d94d5b

### Resource availability

#### Lead contact

Further information and requests for resources should be directed to and will be fulfilled by the lead contact, Dr. Gregory Offer (gregory.offer@imperial.ac.uk).

#### Materials availability

This study did not generate new unique reagents.

#### Data and code availability

This paper analyzes existing, publicly available data. All data is available in the main text or the supplementary information or its origin has been clearly stated. Any additional information required to reanalyze the data reported in this paper are available from the lead contact upon request.

### Method details

For this study, a battery pack with an energy density of 240 Wh·kg^-1^ was assumed, with a design similar to the Tesla Model S NCA pack. Assessed battery chemistries include NCA, NMC622, NMC811, LMO and LFP. The system boundary of this study is illustrated in Figure S1. The techno-economic model takes into account the transportation of battery packs from a collection site in the UK to the respective recycling facility in the UK, the US, Belgium, China, and South Korea. The collection site and recycling locations have been selected to provide a range of transportation distances and reference recycling costs that are representative for Europe, Asia, and the US.

The packs are disassembled down to cell level at the recycling facility, before being treated via hydrometallurgical, pyrometallurgical or direct recycling processes. In the default hydrometallurgical process modeled in EverBatt (Dai et al., 2019) (Version 2019), cells are discharged and disassembled before being shredded and calcinated. This is followed by a physical separation step, leaching, solvent extraction, and precipitation. The default pyrometallurgical procedure starts with smelting battery cells and subsequently subjects the recovered alloy to a series of processes, i.e. leaching, solvent extraction and precipitation, in order to refine cobalt, nickel, copper and iron. EverBatt defines direct recycling as discharging and disassembling cells and extracting electrolyte, followed by shredding and physical separation. The recovered cathode material is then relithiated, using Li_2_CO_3_. Only open-loop recycling processes are assumed in all cases.

All modules and cells are subjected to recycling, regardless of their State-of-Health. For the recycling facility, a yearly throughput of 8,000 tonnes of battery cells was assumed, similar to the one for the SungEel recycling facility in South Korea. Recovered materials from the cells such as nickel, cobalt, copper, aluminum and graphite are then resold, depending on the recycling method and the pack materials (steel, copper, aluminum). Cell and pack manufacturing from recovered materials are excluded in this study. For better comparability, battery fees to the recycler for taking the batteries are not taken into account, as they might differ between countries.

The net recycling profit per kg cells (*NRP*_*kg*_, in $·kg^-1^) is composed of transportation costs (*C*_*T,kg*_), disassembly costs (*C*_*D,kg*_), recycling costs (*C*_*R,kg*_) and the revenue generated from the reselling of recovered materials from the cells (*R*_*cell,kg*_) and packs (*R*_*pack,kg*_) (Equation 1). All values are given per kg cells. To obtain the net recycling profit per kWh (*NRP*_*kWh*_, in $·kWh^-1^), *NRP*_*kg*_ is divided by the energy density of the battery pack (*E*_*batt*_, in Wh·kg^-1^) (Equation 2).

(Equation 1) $$NRP_{kg}=-\left(C_{T,kg}+C_{D,kg}+C_{R,kg}-R_{cell,kg}-R_{pack,kg}\right)$$

(Equation 2) $$NRP_{kWh}=\frac{NRP_{kg}}{E_{batt}}$$

*C*_*T,kg*_ was derived from several shipping agency quotes such as DHL (myDHLi Quote, n.d.), where it was assumed that shipping containers with a weight of 20 tonnes per container are shipped from Coventry, UK, to the respective recycling sites. The recycling sites in the US, China, South Korea and Belgium were chosen based on existing recycling facilities, as shown in Figure 1. The transportation distances from the UK to existing recycling sites in China, South Korea, the US, and Belgium were estimated at 9,750 km, 9,200 km, 5,780 km, and 700 km from the collection point in Coventry. The distance to the recycling site in the UK, which was placed in the West Midlands, was estimated at 35 km from Coventry. As of 2020, the quoted prices were 4,400 $·container^-1^ for shipping to the US, and 3,520 $·container^-1^ to China and South Korea including a 10% surcharge for handling hazardous goods, which is based on experience values from previous battery shipping quotes. The transportation costs of containers, with a weight of 20 tonnes per container, to Belgium and the recycling site in the UK were estimated to be $1,100. Table S8 summarizes the transportation costs in $·tonne^-1^·km^-1^, based on the quoted costs and on the EverBatt transportation cost input values. Note that for the transportation cost based on EverBatt, estimations for transport distances on sea and on land were applied. EverBatt cost values for hazardous goods were used for cost calculations.

The disassembly cost per pack (*C*_*D,pack*_) is calculated, taking into account the labor cost (*C*_*labor*_) at the respective disassembly location as well as the disassembly time (*t*_*D,n*_) and instruction time (*t*_*I,n*_) per step *n* (Equation 3). *t*_*D,n*_ and *t*_*I,n*_ equal 15 sec and 10 sec, based on a study by Das et al.(Das et al., 2000). The disassembly process is derived from the pack layout and publicly available teardown processes (An Extremely Detailed Look At The Porsche Taycan's Engineering Designed To Take On Tesla, n.d.; Everything You Need To Know About Tesla's Lithium-Ion Batteries, n.d.; Tesla Model S Battery Teardown, 2017; The technology behind the new Porsche Taycan, n.d.; WeberAuto, 2019). Disassembly actions that are performed on the entire pack (e.g. taking off the pack cover) are counted as one step, actions that occur on module level are multiplied by the number of modules in the pack. The basic disassembly steps include removing the cover, disconnecting electronics and cables, removing coolant hoses, taking out and opening the modules and removing the cells (Table S3) (Alfaro-Algaba and Ramirez, 2020; Wegener et al., 2015). Note that the actual cell separation step has not been included. It is assumed that the disassembly process is carried out manually with tools (no automated processes) (Harper et al., 2019; Wegener et al., 2014). The disassembly cost per kg cell is calculated from *C*_*D,pack*_ divided by the sum of the cell mass (*m*_*cell*_) in the pack (Equation 4). The disassembly cost per kWh is obtained by from *C*_*D,pack*_ divided by the energy density of the pack (*E*_*batt*_) (Equation 5). Please note that the order and number disassembly steps are estimations, in order to obtain a cost estimate.

(Equation 3) $$C_{D,pack}=C_{labour}\cdot \sum_{n=1}^{N}\left(t_{D,n}+t_{I,n}\right)$$

(Equation 4) $$C_{D,kg}=\frac{C_{D,pack}}{\sum_{j=1}^{J}m_{cell,j}}$$

(Equation 5) $$C_{D,kWh}=\frac{C_{D,pack}}{E_{batt}}$$

*C*_*R,kg*_ for recycling facilities in China, South Korea, and US is calculated according to Equation 6 using EverBatt (Dai et al., 2019). EverBatt calculates labor costs (*C*_*labor*_), material costs (*C*_*material*_) for the respective recycling method, utility costs (*C*_*utilities*_) such as electricity, water, natural gas, and further general expenses (*C*_*general*_), such as maintenance and operating costs, taxes, rent, administrative costs, and R&D, based on its input data (Dai et al., 2019). The costs are divided by the yearly throughput of battery cells (here 8,000 tonnes) to obtain the recycling cost per kg cells. To obtain the recycling cost per kWh, *C*_*R,kg*_ is divided by *E*_*batt*_ (Equation 7). A detailed description of the EverBatt model and its input parameters for China, South Korea and US can be found in the program documentation (Dai et al., 2019). The labor costs for the US, China and South Korea amount to 20 $·hr^-1^, 3 $·hr^−-1^, and 10 $·hr^-1^, respectively.

Since no European recycling location is included in EverBatt at the time of publishing, Umicore in Belgium and a hypothetical recycling facility in the UK were chosen as representatives for European recycling facilities. Geographically specific costs (e.g. water, electricity) for Belgium and the UK were obtained from publicly available data and included in the EverBatt model. The hourly labor cost for Belgium (48.1 $·hr^-1^) and the UK (33.85 $·hr^-1^) are based on 2019 values given in (Kolakovic and Lethyienne, 2020).

(Equation 6) $$C_{R,kg}=\;\frac{C_{labour}+C_{materials}+C_{utilities}+C_{general}}{throughput}$$

(Equation 7) $$C_{R,kWh}=\frac{C_{R,kg}}{E_{batt}}$$

*R*_*cell,kg*_ refers to the revenue generated from reselling recovered cell materials. Depending on the recycling method, materials include copper, aluminum, nickel, cobalt, manganese, or the cathode material in its original structure (for direct recycling). The recovering efficiencies for the three recycling processes are summarized in Table S9, as given in EverBatt. *R*_*cell,kg*_ is calculated according to Equation 8, taking into account the mass of recovered material *m*_*i*_ with its unit price *up*_*i*_ as given in EverBatt (Dai et al., 2019). The cost of disposing of unrecycled material in landfills is taken into account in the EverBatt model. *R*_*pack,kg*_ refers to revenue generated from the reselling of pack casing material, such as copper, steel, and aluminum. It is calculated in the same manner as *R*_*cell,kg*_, where the mass of recoverable pack material *m*_*i*_ for the respective pack design is multiplied with the unit price, *up*_*i*_, of the respective material (Equation 10). The amount and value of recoverable pack material is based on values given by BatPaC (Nelson et al., 2019) (Version 4.0 released in 2020). To obtain the revenue in kWh units, *R*_*cell,kg*_ and *R*_*pack,kg*_ are divided by the energy density of the pack (*E*_*batt*_) (Equations (Equation 9), (Equation 11)). Since the recovered materials are regarded as a global commodity, the same prices and thus revenues are assumed for all locations (Dai et al., 2019).

(Equation 8) $$R_{cell,kg}=\sum_{i}m_{i}\cdot up_{i}$$

(Equation 9) $$R_{cell,kWh}=\frac{R_{cell,kg}}{E_{batt}}$$

(Equation 10) $$R_{pack,kg}=\sum_{i}m_{i}\cdot up_{i}$$

(Equation 11) $$R_{pack,kWh}=\frac{R_{pack,kg}}{E_{batt}}$$

## Footnotes

Supplemental information can be found online at https://doi.org/10.1016/j.isci.2021.102787.
