# Supplementary material for: Financial viability of electric vehicle lithium-ion battery recycling
Source: iScience. 2021 Jun 25;24(7):102787. doi: 10.1016/j.isci.2021.102787 (PMC8283134; doi:10.1016/j.isci.2021.102787)
Supplement: Document S1. Figures S1–S10 and Tables S1–S9 [file mmc1.pdf]

iScience, Volume *24*

Supplemental information

Financial viability of electric vehicle lithium-ion battery recycling

Laura Lander, Tom Cleaver, Mohammad Ali Rajaeifar, Viet Nguyen-Tien, Robert J.R. Elliott, Oliver Heidrich, Emma Kendrick, Jacqueline Sophie Edge, and Gregory Offer

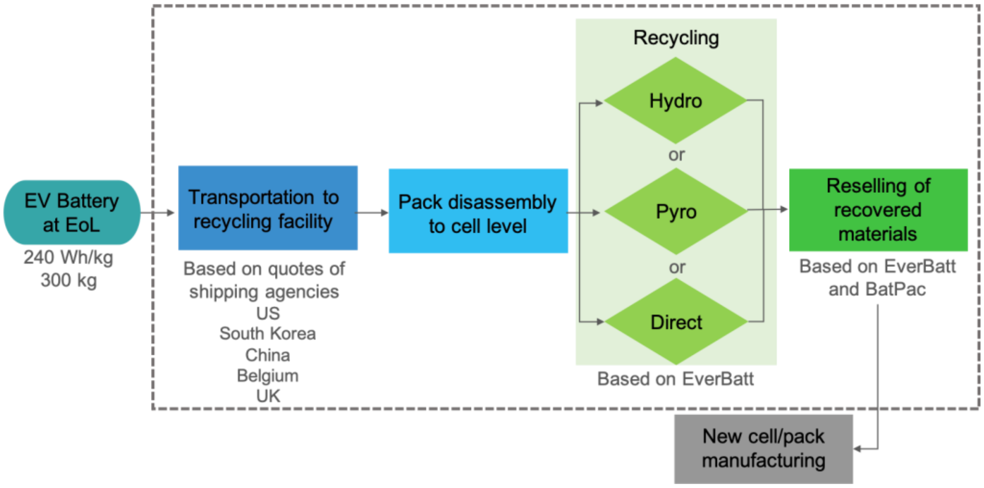

Figure S1. Techno-economic model, related to STAR Methods.
System boundaries of the techno-economic analysis.

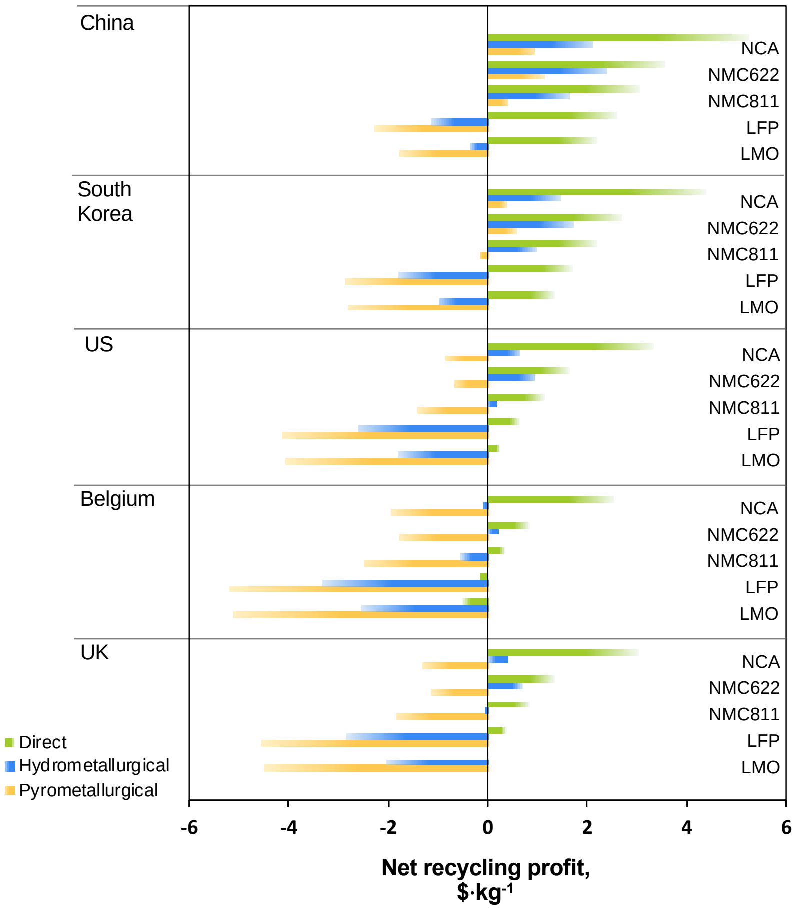

Figure S2. Net recycling profits, related to Figure 2.
Net recycling profit in $\$\cdot kg^{-1}$ for battery packs with different cathode chemistries, using pyrometallurgical, hydrometallurgical or direct recycling processes for the selected countries.

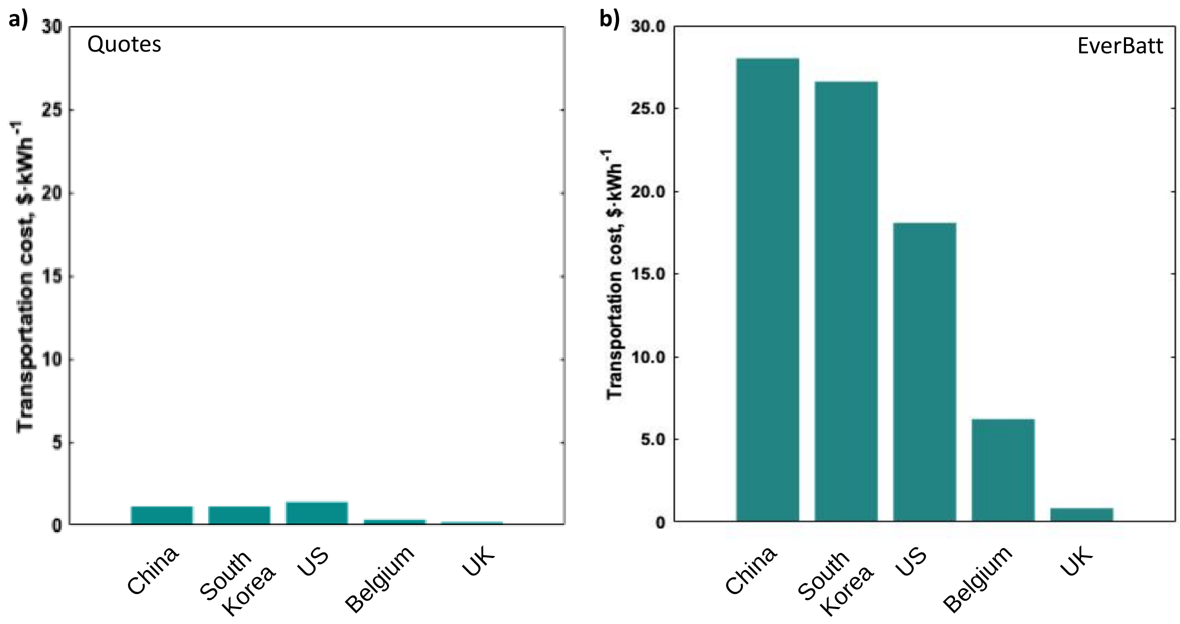

Figure S3. Transportation costs, related to STAR Methods and Figure 3.
Transportation cost given in $·kWh^{-1} for a Tesla Model S battery pack shipped to the selected countries based on a) quotes (given in 2020) and b) EverBatt shipping fees.

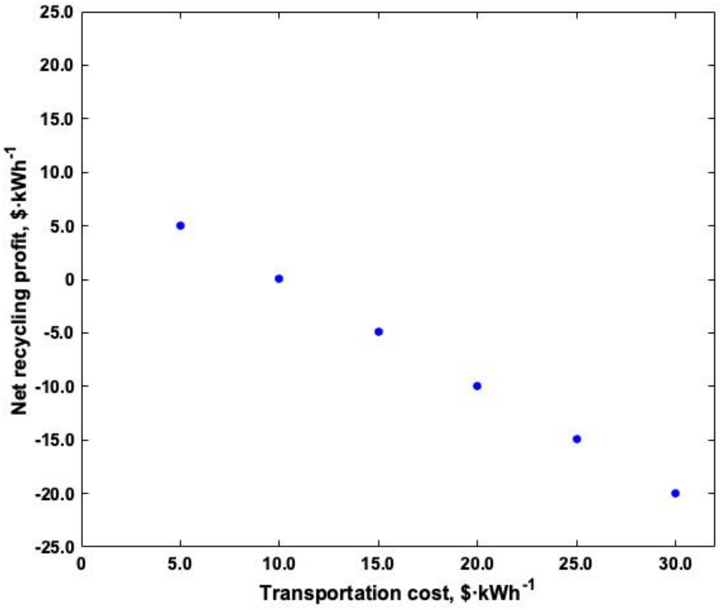

Figure S4. Transportation cost sensitivity analysis, related to STAR Methods and Section 1.1.
Evolution of net recycling profit in function of transportation costs for an NCA battery pack treated via hydrometallurgical recycling in China.

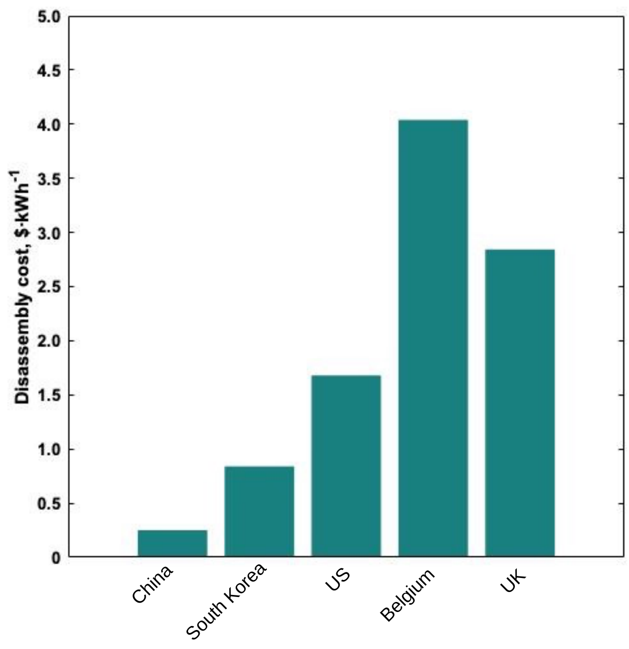

Figure S5. Disassembly cost, related to STAR Methods.

Disassembly cost, given in $\$\cdot kWh^{-1}$ for a Tesla Model S battery pack dismantled in the selected countries.

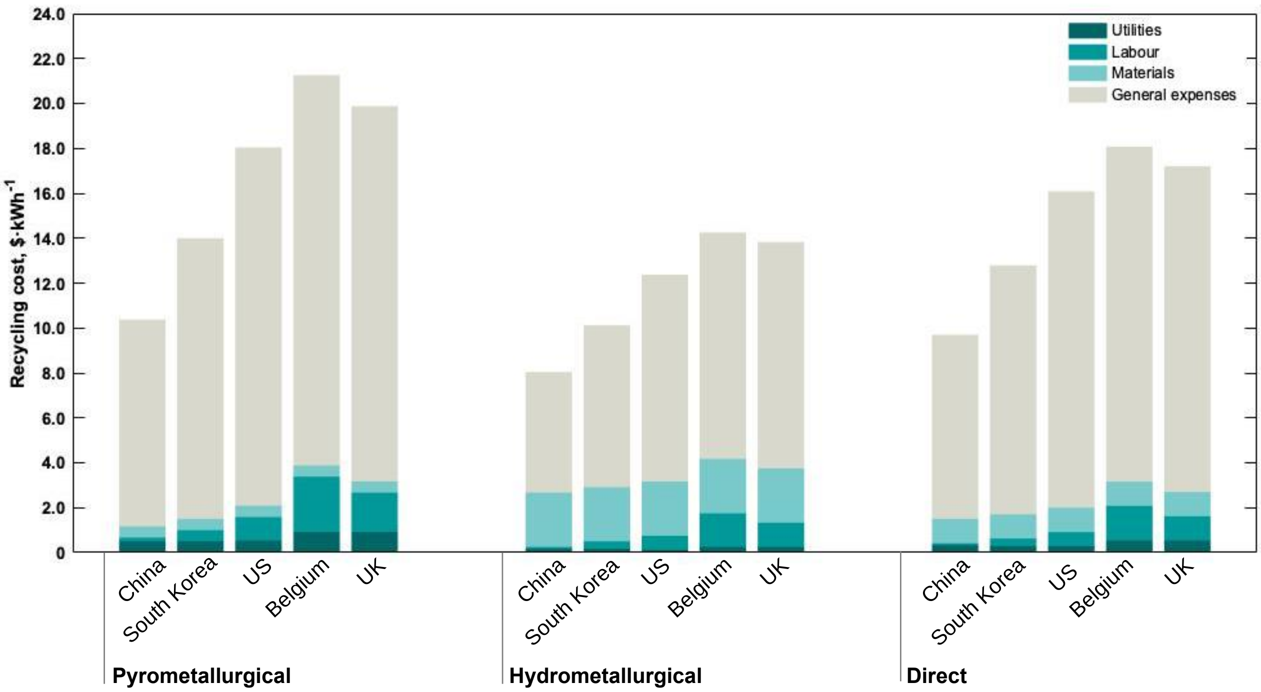

Figure S6. Recycling cost breakdown, related to STAR Methods.

Breakdown of the recycling process costs ($\$\cdot kWh^{-1}$) into utility costs (i.e. electricity, water, natural gas), labour costs, materials cost, and general expenses (i.e. operating and maintenance costs, rent, insurance, taxes, etc.) for a 240 $Wh\cdot kg^{-1}$ NCA battery pack.

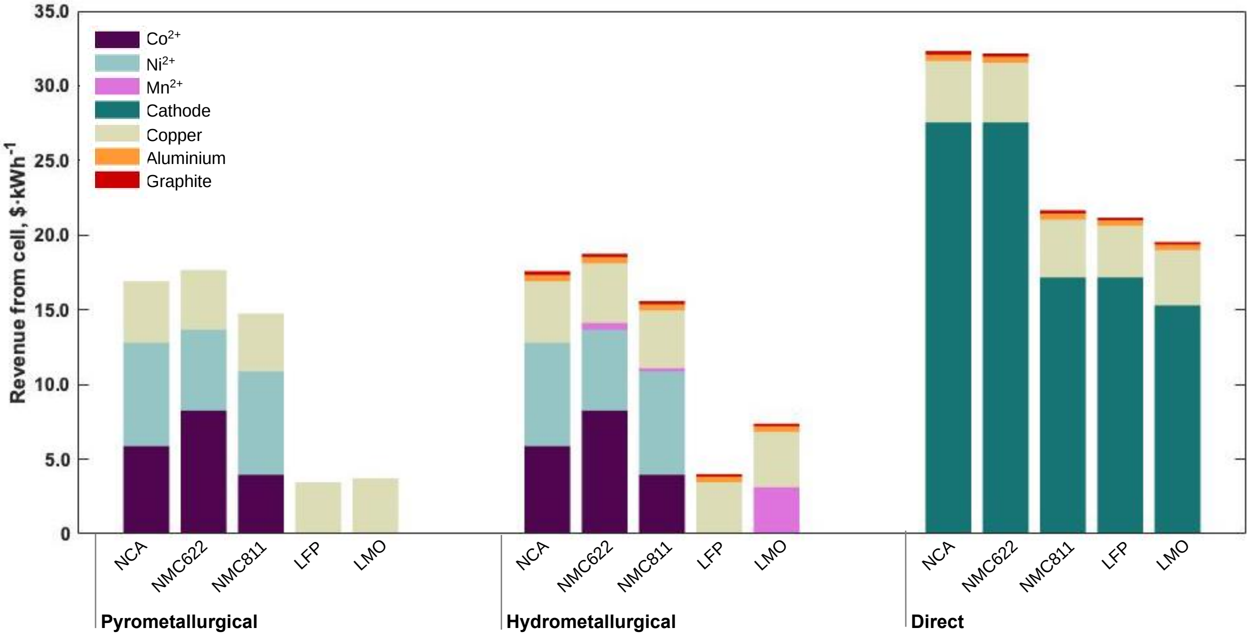

Figure S7. Revenue from recycling, related to STAR Methods.
Revenue generated from recycled cell materials for NCA, NMC622, NMC811, LFP, and LMO battery packs.

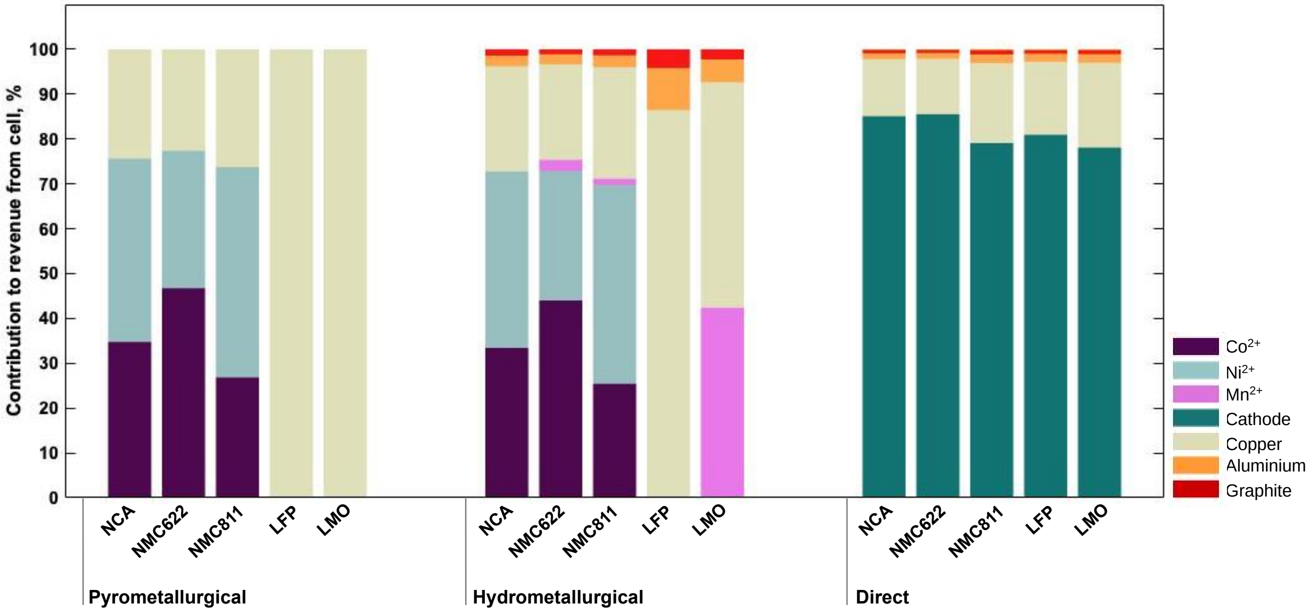

Figure S8. Revenue contribution from recycled materials, related to STAR Methods.
Contribution (in %) of the recovered materials to the overall revenue.

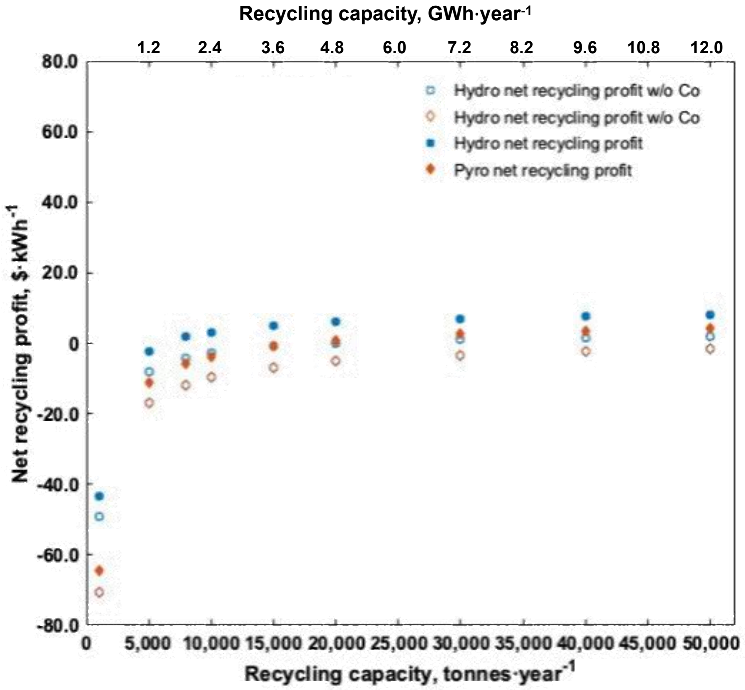

Figure S9. Economies of scale, related to Figure 4.

Net recycling profit as a function of the yearly recycling capacity for pyrometallurgical and hydrometallurgical recycling in the UK for a 240 Wh·kg^{-1} NCA battery pack with and without revenue generated from Co.

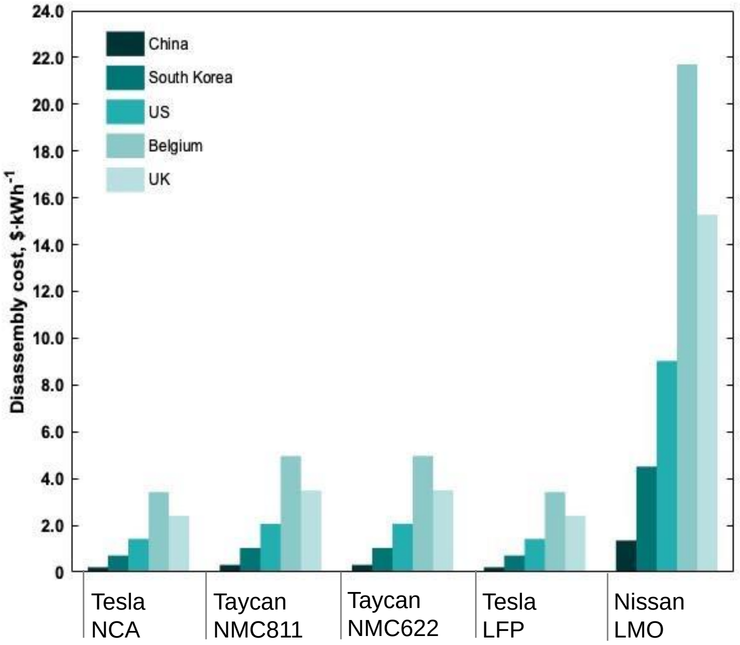

Figure S10. Disassembly cost, related to STAR Methods.

Disassembly cost given in $·kWh^{-1} for commercial battery packs dismantled in the selected countries.

Table S1. Net recycling profits, related to Figure 2 and STAR Methods.

Net recycling profit in $\$\cdot kWh^{-1}$ for battery packs with different cathode chemistries, using pyrometallurgical, hydrometallurgical or direct recycling processes for China, South Korea, the US, Belgium, and the UK.

		**Pyrometallurgical** ($\$\cdot kWh^{-1}$)	**Hydrometallurgical** ($\$\cdot kWh^{-1}$)	**Direct** ($\$\cdot kWh^{-1}$)
**China**	**NCA**	3.94	8.66	21.90
	**NMC622**	4.69	9.83	14.86
	**NMC811**	1.77	6.66	12.77
	**LFP**	-9.52	-4.92	10.77
	**LMO**	-7.39	-1.55	9.15
**South Korea**	**NCA**	1.60	6.10	18.27
	**NMC622**	2.35	7.27	11.23
	**NMC811**	-0.56	4.10	9.15
	**LFP**	-11.90	-7.48	7.15
	**LMO**	-11.65	-4.10	5.52
**US**	**NCA**	-3.59	2.75	13.83
	**NMC622**	-2.88	3.91	6.79
	**NMC811**	-5.84	0.75	4.70
	**LFP**	-17.13	-10.84	2.70
	**LMO**	-16.88	-7.46	1.08
**Belgium**	**NCA**	-8.07	-0.32	10.55
	**NMC622**	-7.32	0.84	3.51
	**NMC811**	-10.24	-2.32	1.43
	**LFP**	-21.57	-13.91	-0.57
	**LMO**	-21.36	-10.53	-2.20
**UK**	**NCA**	-5.42	1.75	12.66
	**NMC622**	-4.71	2.91	5.62
	**NMC811**	-7.63	-0.25	3.54
	**LFP**	-18.92	-11.84	1.54
	**LMO**	-18.75	-8.46	-0.09

Table S2. Net recycling profits, related to Figure 3 and STAR Methods.

Net recycling profit in $\$\cdot kWh^{-1}$ assuming transportation costs as given in EverBatt.

		**Pyrometallurgical** ($\$\cdot kWh^{-1}$)	**Hydrometallurgical** ($\$\cdot kWh^{-1}$)	**Direct** ($\$\cdot kWh^{-1}$)
**China**	**NCA**	-22.84	-17.97	-4.88
	**NMC622**	-22.09	-16.80	-11.92
	**NMC811**	-25.01	-19.97	-14.01
	**LFP**	-36.30	-31.55	-16.01
	**LMO**	-34.17	-28.17	-17.63
**South Korea**	**NCA**	-23.78	-19.28	-7.11
	**NMC622**	-23.03	-18.11	-14.15
	**NMC811**	-25.95	-21.28	-16.24
	**LFP**	-37.28	-32.86	-18.24
	**LMO**	-37.03	-29.49	-19.86
**US**	**NCA**	-20.12	-13.78	-2.70
	**NMC622**	-19.41	-12.62	-9.74
	**NMC811**	-22.37	-15.78	-11.83
	**LFP**	-33.66	-27.37	-13.83
	**LMO**	-33.41	-23.99	-15.45
**Belgium**	**NCA**	-13.91	-6.16	4.76
	**NMC622**	-13.16	-4.99	-2.28
	**NMC811**	-16.07	-8.16	-4.37
	**LFP**	-27.41	-19.74	-6.37
	**LMO**	-27.20	-16.37	-7.99
**UK**	**NCA**	-5.78	1.72	12.43
	**NMC622**	-5.03	2.89	5.39
	**NMC811**	-7.94	-0.28	3.31
	**LFP**	-19.36	-11.86	1.31
	**LMO**	-19.015	-8.48	-0.32

Table S3. Battery pack disassembly – Tesla, related to STAR Methods.
Disassembly steps for a Tesla Model S battery pack.

Step number	Disassembly step
1	Peel off glued plastic cover
2	Unscrew top cover
3	Unclamp top cover
4	Empty coolant
5	Disconnect wires
6	Disconnect BMS
7	Disconnect coolant hoses
8-23	Unscrew modules from tray
24-39	Take off module covers
40-55	Take out busbars
56-71	Take out coolant circuit
72-87	Remove cells

Table S4. Battery pack disassembly – Porsche, related to STAR Methods.
Disassembly steps for a Porsche Taycan battery pack.

Step number	Disassembly step
1	Unscrew pack cover
2	Unscrew upper parts
3	Disconnect bus bars
4	Disconnect electric parts
5	Take apart welded battery frame
6	Take off bus bars
7-39	Take out modules
40-72	Open modules
73-105	Take out coolant hoses
106-138	Remove cells

Table S5. Battery pack disassembly – Nissan, related to STAR Methods.
Disassembly steps for a Nissan Leaf battery pack.

Step number	Disassembly step
1	Unscrew top cover
2	Disconnect BMS
3	Take out junction block
4	Disconnect electrical connector
5	Disconnect heater controller
6	Disconnect temperature sensors
7	Take out brackets
8	Take out structural support
9	Disconnect cables
10-57	Unscrew modules from battery tray
58	Take out brackets
59	Take out spacers
60	Cut electrical connections
61-108	Open housing of modules
109-156	Remove cells

Table S6. Battery pack specifics, related to Figure 5.
Specifics for various commercial EV battery packs.

	Tesla Model S	Porsche Taycan NMC622	Porsche Taycan NMC811	Tesla Model S LFP	Nissan Leaf
Total pack weight (kg)	540	630	553	1009	295
Weight cells only (kg)	319	382	305	788	151
Number of modules	16	33	33	16	48
Number of cells	7104	396	396	10,368	192
Cell weight (kg)	0.045	0.965	0.772	0.076	0.785
Energy (kWh)	85	93	93	85	24
Energy density (Wh/kg)	266	243	304	108	159

Table S7. Net recycling profits, related to Figure 5 and STAR Methods.

Net recycling profit in $\$\cdot kWh^{-1}$ for different commercial battery designs using pyrometallurgical, hydrometallurgical or direct recycling processes for China, South Korea, the US, Belgium, and the UK.

		**Pyrometallurgical** ($\$\cdot kWh^{-1}$)	**Hydrometallurgical** ($\$\cdot kWh^{-1}$)	**Direct** ($\$\cdot kWh^{-1}$)
**China**	**Tesla NCA**	3.56	7.95	19.74
	**Taycan NMC622**	4.39	9.61	14.42
	**Taycan NMC811**	1.06	5.04	9.74
	**Tesla LFP**	-20.42	-9.85	24.72
	**Nissan LMO**	-15.18	-3.29	12.63
**South Korea**	**Tesla NCA**	1.49	5.54	16.50
	**Taycan NMC622**	1.95	6.79	10.70
	**Taycan NMC811**	-1.04	2.63	6.60
	**Tesla LFP**	-24.91	-15.08	17.46
	**Nissan LMO**	-21.05	-9.66	4.88
**US**	**Tesla NCA**	-3.14	2.56	12.55
	**Taycan NMC622**	-3.41	3.29	6.12
	**Taycan NMC811**	-5.60	-0.40	2.72
	**Tesla LFP**	-35.71	-21.72	8.41
	**Nissan LMO**	-32.27	-18.05	-5.15
**Belgium**	**Tesla NCA**	-7.05	-0.07	9.76
	**Taycan NMC622**	-8.39	-0.34	2.33
	**Taycan NMC811**	-10.05	-3.80	-0.81
	**Tesla LFP**	-42.49	-25.43	4.32
	**Nissan LMO**	-47.89	-31.54	-18.89
**UK**	**Tesla NCA**	-4.65	2.10	11.75
	**Taycan NMC622**	-5.40	2.40	4.87
	**Taycan NMC811**	-7.36	-1.31	1.52
	**Tesla LFP**	-38.23	-21.55	7.74
	**Nissan LMO**	-39.26	-23.15	-10.82

Table S8. Transportation costs, related to STAR Methods.

Comparison of transportation costs in $\$\cdot tonne^{-1}\cdot km^{-1}$ based on EverBatt transportation cost input values and as obtained from shipping agency quotations.

	China	South Korea	US	Belgium	UK
**Estimated direct line distance to recycling site** (in km)	9,750	9,200	5,780	700	35
**Quoted cost per 20 t container** (in $\$\cdot container^{-1}$)	3,520	3,520	4,400	1,100*	1,100*
**Cost** (in $\$\cdot tonne^{-1}\cdot km^{-1}$)	0.018	0.019	0.04	0.08	1.58
**Cost using EverBatt values** (in $\$\cdot tonne^{-1}\cdot km^{-1}$)	0.41	0.41	0.45	1.26*	3.35*

*Own estimations

Table S9. Recycling recovery efficiencies, related to STAR Methods.

Default recovery efficiencies for pyrometallurgical, hydrometallurgical and direct recycling as given in EverBatt.

	Pyrometallurgical	Hydrometallurgical	Direct Physical
**Copper**	90%	90%	90%
**Steel**	90%	90%	90%
**Aluminum**	-	90%	90%
**Graphite**	-	90%	90%
**Plastics**	-	50%	50%
**Li^{+} in product**	-	90%	-
**LCO**	-	-	90%
**NMC(111)**	-	-	90%
**NMC(622)**	-	-	90%
**NMC(811)**	-	-	90%
**NCA**	-	-	90%
**LMO**	-	-	90%
**LFP**	-	-	90%
**Co^{2+} in product**	98%	98%	-
**Ni^{2+} in product**	98%	98%	-
**Mn^{2+} in product**	-	98%	-
**Electrolyte Organics**	-	50%	50%
